# Paramagnetic Metal Accumulation in the Deep Gray Matter Nuclei Is Associated With Neurodegeneration in Wilson’s Disease

**DOI:** 10.3389/fnins.2020.573633

**Published:** 2020-09-16

**Authors:** Xiang-Zhen Yuan, Gai-Ying Li, Jia-Lin Chen, Jian-Qi Li, Xiao-Ping Wang

**Affiliations:** ^1^Department of Neurology, Tongren Hospital, Shanghai Jiao Tong University School of Medicine, Shanghai, China; ^2^Shanghai Key Laboratory of Magnetic Resonance, School of Physics and Electronic Science, East China Normal University, Shanghai, China

**Keywords:** Wilson’s disease, quantitative susceptibility mapping, iron, copper, neurodegeneration

## Abstract

**Background:**

Neuropathological studies have revealed copper and iron accumulation in the deep gray matter (DGM) nuclei of patients with Wilson’s disease (WD). However, the association between metal accumulation and neurodegeneration in WD has not been well studied *in vivo*. The study was aimed to investigate whether metal accumulation in the DGM was associated with the structural and functional changes of DGM in neurological WD patients.

**Methods:**

Seventeen neurological WD patients and 20 healthy controls were recruited for the study. Mean bulk susceptibility values and volumes of DGM were obtained from quantitative susceptibility mapping (QSM). Regions of interest including the head of the caudate nucleus, globus pallidus, putamen, thalamus, substantia nigra, red nucleus, and dentate nucleus were manually segmented. The susceptibility values and volumes of DGM in different groups were compared using a linear regression model. Correlations between susceptibility values and volumes of DGM and Unified Wilson’s Disease Rating Scale (UWDRS) neurological subscores were investigated.

**Results:**

The susceptibility values of all examined DGM in WD patients were higher than those in healthy controls (*P* < 0.05). Volume reductions were observed in the head of the caudate nucleus, globus pallidus, putamen, thalamus, and substantia nigra of WD patients (*P* < 0.001). Susceptibility values were negatively correlated with the volumes of the head of the caudate nucleus (*r*_*p*_ = −0.657, *P* = 0.037), putamen (*r*_*p*_ = −0.667, *P* = 0.037), and thalamus (*r*_*p*_ = −0.613, *P* = 0.046) in WD patients. UWDRS neurological subscores were positively correlated with the susceptibility values of all examined DGM. The susceptibility values of putamen, head of the caudate nucleus, and dentate nucleus could well predict UWDRS neurological subscores.

**Conclusion:**

Our study provided *in vivo* evidence that paramagnetic metal accumulation in the DGM was associated with DGM atrophy and neurological impairment. The susceptibility of DGM could be used as a biomarker to assess the severity of neurodegeneration in WD.

## Introduction

Wilson’s disease (WD), also known as hepatolenticular degeneration, is a copper-overload inherited disease caused by *ATP7B* mutations on chromosome 13. *ATP7B* encodes a copper-transporting P-type ATPase ATP7B, which plays a critical role in maintaining copper homeostasis (Czlonkowska et al., 2018). The dysfunction of ATP7B causes excessive copper accumulation in the liver, and then large amount of non-ceruloplasmin-bound copper is released into the circulation, leading to secondary copper accumulation in the brain, kidney, and other organs (Bandmann et al., 2015). Liver disease and neurological symptoms are the most common clinical features of WD. Copper chelators can effectively improve the hepatic symptoms of WD patients, but they are less effective in improving neurological symptoms and even cause neurological deterioration in initial treatment (Li et al., 2016; Czlonkowska and Litwin, 2017). The pathogenesis of neurological impairment in WD is still not clear. In addition to the toxic effects of copper on the central nervous system, iron accumulation may also aggravate neural damage (Dusek et al., 2015, 2017).

Magnetic resonance imaging (MRI) is the most sensitive imaging tool in the diagnosis of WD. Abnormal signals were observed in more than 90% of the neurological WD patients (Litwin et al., 2013a). The most common brain MRI findings are symmetrical or asymmetric hyperintensities in T2-weighted images in the deep gray matter (DGM) nuclei and brain stem. Hyperintensities in T2-weighted images in the brain may reflect neuropathological changes such as edema, demyelination, and gliosis, which are largely reversible after effective anti-copper treatment (King et al., 1996; Czlonkowska et al., 2018). Brain atrophy is another common imaging feature of neurological WD patients, and both DGM and white matter are affected. Recent studies suggested that total brain volume and the volumes of white matter and gray matter were associated with the severity of neurological impairment in WD patients (Smolinski et al., 2019; Dusek et al., 2020). However, it is not clear whether this correlation still exists in single DGM nuclei. Although WD is a copper-overload disease, the correlation between copper accumulation and neuropathological changes is still controversial (Poujois et al., 2017). An increasing number of studies indicate that iron accumulation in the DGM is involved in the neuropathological changes of WD. Dusek et al. (2017) reported that increased iron content was detected in the basal ganglia of WD patients, and severe putamen (Put) pathological changes were associated with an increased number of iron-containing macrophages. In addition, hypointensities in T2/T2^∗^-weighted images and susceptibility-weighted images (SWI) in the DGM are frequently present in WD patients, also reflecting paramagnetic metal (mainly iron) accumulation in these regions (Skowronska et al., 2013; Dusek et al., 2017). A recent MRI- histopathological study demonstrated that R2^∗^ values correlated with iron concentration in the DGM of WD patients (Dusek et al., 2017). However, R2^∗^ mapping cannot distinguish between iron and diamagnetic substances. R2^∗^ mapping also depends on factors such as field strength, homogeneity of iron distribution, and suffers from blooming artifacts (Bilgic et al., 2012).

Quantitative susceptibility mapping (QSM) is a recently developed MRI post-processing technique, which can quantitatively measure tissue susceptibility and reflect the concentrations of paramagnetic metal *in vivo* (Wang et al., 2017; Li et al., 2018). Using QSM, several recent studies found significantly increased susceptibility in the DGM of neurological WD patients, suggesting paramagnetic metal accumulation in the DGM is a character of neuropathology (Fritzsch et al., 2014; Doganay et al., 2018; Dezortova et al., 2019). In addition, QSM performed better than R2^∗^ in distinguishing WD from healthy individuals and showed excellent diagnostic accuracy in the diagnosis of WD (Li et al., 2020).

Intracellular ferritin and hemosiderin are considered as the main source of the DGM susceptibility, which has been confirmed in recent MRI-histopathology studies (Langkammer et al., 2012; Hametner et al., 2018). *In vitro* evidences indicate that the Cu^2+^ compounds are slightly paramagnetic and the Cu^+^ compounds are diamagnetic, but the magnetic features of copper *in vivo* are still unclear (Valdes Hernandez Mdel et al., 2012). Considering that a large amount of copper accumulates in the brains of WD patients, increased susceptibility in the DGM may be explained by copper and iron accumulation.

Several case reports reported that metal accumulation in the DGM changed with the progression or improvement of neurological symptoms (Dusek et al., 2018; Zaino et al., 2019). It is speculated that metal accumulation in the DGM is a surrogate indicator of oxidative stress and neuronal loss (Yan et al., 2018). However, the relationship between metal accumulation and neurodegeneration in WD has not been quantitatively studied *in vivo*. Moreover, a quantitative biomarker for the evaluation of neurodegeneration in WD is still lacking. Validated neuroimaging biomarker will be very helpful in treatment monitoring and outcome prediction.

In this study, we hypothesized that metal accumulation in the DGM was associated with the structural and functional changes of DGM in neurological WD patients. Using the recently developed MRI technique QSM, we quantitatively measured the susceptibility values of DGM, and investigated the association between metal accumulation and DGM volume changes. Furthermore, the association between metal accumulation and the severity of neurological impairment in WD was also assessed.

## Materials and Methods

### Subjects

Twenty neurological WD patients were recruited from the Department of Neurology, Tongren Hospital, Shanghai Jiao Tong University School of Medicine, between December 2018 and December 2019. WD was confirmed according to the Leipzig diagnostic criteria, including low serum ceruloplasmin, presence of Kayser-Fleischer rings on slit-lamp examination, high 24-hour urine copper excretion, and genetic confirmation (Ferenci et al., 2003). All WD patients were on anti-copper treatment with D-penicillamine or dimercaptosuccinic acid (DMSA). Before MRI scans, the severity of neurological impairment in WD patients was assessed by two neurologists with consensus using Unified Wilson’s Disease Rating Scale (UWDRS) neurological subscale (27 items, 208 points) (Leinweber et al., 2008; Volpert et al., 2017). Neurological WD was defined as the presence of neurological symptoms at the time of onset or anytime during the course of the disease. Neurological WD patients aged 18 years or older were included in this study. Exclusion criteria included severe neurological impairment or severe liver decompensation, history of other neuropsychiatric disorders or liver disease, and hepatic encephalopathy. T1 hyperintensities in the lentiform nucleus of WD patients are associated with portosystemic shunting and hepatic encephalopathy (Kozic et al., 2003), which may affect regional susceptibility duo to manganese accumulation and the evaluation of neurological symptoms (Lee et al., 2018). Therefore, WD patients with T1 hyperintensities in the lentiform nucleus were excluded from the study. Twenty age and gender matched healthy controls (HC) were recruited for the study. This study was approved by the Medical Ethics Committee of Tongren Hospital, Shanghai Jiao Tong University School of Medicine. All subjects were informed about the study and signed written consent before participation.

### Imaging Data Acquisition and Procession

Magnetic resonance imaging scans were carried out on a clinical 3T MRI system (Magnetom Prisma Fit, Siemens Healthcare, Erlangen, Germany) equipped with a 20-channel head coil. The susceptibility maps were acquired from a 3D spoiled multi-echo gradient-echo (GRE) sequence with the following imaging parameters: repetition time (TR) = 31 ms, the first echo time (TE1) = 4.07 ms, echo spacing (ΔTE) = 4.35 ms, numbers of echoes = 6, flip angle = 12°, field of view (FOV) = 240 mm× 200 mm, in-plane resolution = 0.83 mm× 0.83 mm, slice thickness = 0.8 mm, number of slices = 192, acquisition time = 7.22 min. A generalized auto-calibrating partially parallel acquisition (GRAPPA) with an acceleration factor of two in the right-left direction and elliptical sampling were used to reduce the acquisition time. T1-weighted, T2-weighted, and T2-weighted fluid-attenuated inversion recovery (FLAIR) images were also obtained from all subjects. T1-weighted images were acquired using a magnetization prepared rapid gradient echo (MPRAGE) sequence with TR = 2530 ms, inversion time (TI) = 1100 ms, TE = 2.98 ms, FOV = 256 mm× 256 mm, matrix = 256 × 256, slice thickness = 1 mm, slice number = 192, acquisition time = 6.03 min. T2-weighted images were acquired using a fast spin echo sequence with TR = 5400 ms, TE = 105 ms, FOV = 220 mm× 220 mm, matrix = 448 × 448, slice thickness = 3 mm, slice number = 35, acquisition time = 1.44 min. T2-weighted FLAIR were obtained with TR = 5500 ms, TI = 1920 ms, TE = 83 ms, FOV = 220 mm× 220 mm, matrix = 320 × 320, slice thickness = 3 mm, slice number = 35, acquisition time = 2.47 min.

T1-weighted, T2-weighted, and FLAIR images were visually assessed by two neurologists (X-ZY and X-PW). Abnormalities in the corpus striatum, thalamus (Th), midbrain, pons, and cerebellum were recorded. Abnormal signal was defined as deviation from the conventionally accepted signal intensity of a given structure. Cortical and cerebellar atrophy was assessed according to the global cortical atrophy (GCA) score (Pasquier et al., 1996). Central atrophy was assessed by measuring the width of the third ventricle. WD patients were considered to have brain atrophy if the GCA score >1 and/or the width of the third ventricle >6 mm (Dusek et al., 2020). When disagreements arose, consensus was reached through discussion.

The susceptibility maps were reconstructed using the Morphology Enabled Dipole Inversion with automatic uniform cerebrospinal fluid (CSF) zero reference (MEDI + 0) algorithm (Liu et al., 2018). The reconstruction procedure involved the following steps: The field map was first estimated by performing a one-dimensional temporal unwrapping of the phase on each voxel followed by a weighted least squares fit of the temporally unwrapped phases in each voxel over TE (Liu et al., 2013). Then, a magnitude image guided spatial unwrapping algorithm was used to account for frequency aliasing on the field map (Cusack and Papadakis, 2002). Thirdly, the background field was removed using the projection onto dipole fields (PDF) method (Liu et al., 2011). Finally, the remaining tissue field was inverted to generate a susceptibility map using MEDI (Liu et al., 2012), and the susceptibility of ventricular CSF was used as zero reference (Straub et al., 2017). The ventricular CSF mask M_*CSF*_ was determined from the brain ROI mask M and the R2^∗^ map by taking advantage of the low R2^∗^ values of CSF and by imposing connectivity (Supplementary Figure S1). The parameters for MEDI + 0 used the default settings in the MEDI toolbox, λ_1_ and λ_2_ were 1000 and 100, respectively. The threshold of R2^∗^ was 5 s^−1^ (Liu et al., 2018).

**FIGURE 1:**
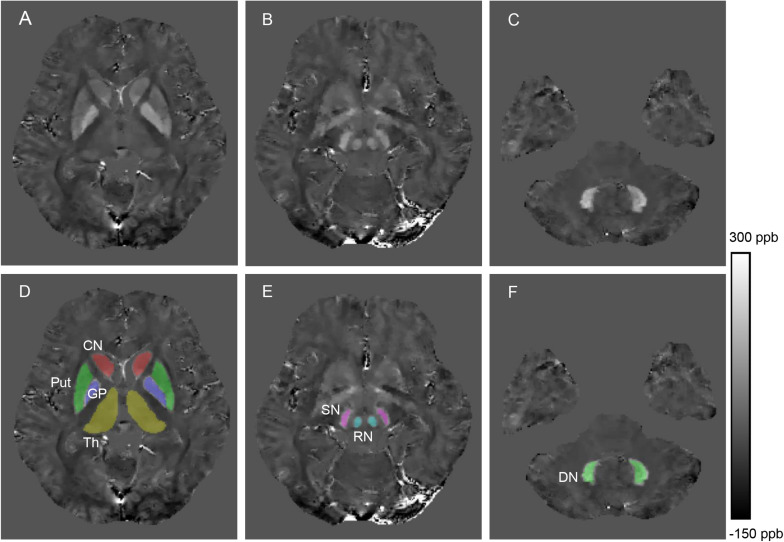
Example images showing segmentation of regions of interest from a healthy control (28 years old, male). The QSM image **(A)** and example segmentations **(D)** include the head of the caudate nucleus (CN), globus pallidus (GP), putamen (Put) and thalamus (Th). The QSM image **(B)** and example segmentations **(E)** include the red nucleus (RN) and substantia nigra (SN). The QSM image **(C)** and example segmentation **(F)** include the dentate nucleus (DN).

The 3D regions of interest (ROIs) including the bilateral head of the caudate nucleus (CN), globus pallidus (GP), Put, Th, substantia nigra (SN), red nucleus (RN), and dentate nucleus (DN), were manually segmented on the susceptibility maps by a rater who was blinded to subject information using ITK-SNAP^1^. ROIs were drawn according to their anatomical boundaries on all sections where the DGM was visible (Figure 1). The most inferior or most superior slice of ROIs were excluded to minimize partial volume effects, and voxels at the boundaries were also excluded. ROIs were confirmed by a senior neurologist (X-PW) with more than 30 years of neuroimaging experience. To account for the effect of head size variability across individuals, the volume of each ROI was normalized with the skull-scaling factor, which was obtained from T1-weighted images using FSL’s SIENAX tool (Smith et al., 2002). Susceptibility values of each ROI in the left and right hemispheres were averaged for further analysis.

### Statistical Analyses

Mann-Whitney *U* test was used to compare the age distribution between WD patients and HC. Susceptibility values and volumes of ROIs in each group were compared using a linear regression model, and age and gender were used as covariates to correct for their potential influence on susceptibility. Correlation analysis was performed between the susceptibility values and volumes of ROIs and UWDRS neurological subscores. Since the data could not completely pass the normality test (Kolmogorov-Smirnov test), Spearman rank correlation coefficients (*r*) were calculated to quantify correlation strength. To control for the effects of age, gender, and disease duration, partial Spearman correlation coefficients (*r*_*p*_) were calculated. The *P*-values of multiple comparisons and correlation analysis were corrected by controlling the false discovery rate (FDR) at a level of 0.05 (Benjamini and Hochberg, 1995). To test whether susceptibility values of DGM could predict the severity of neurological impairment, a multiple regression model with UWDRS neurological subscores as the dependent variable and susceptibility values of DGM as predictor variables was performed. The multiple regression model was performed using a stepwise method, which continuously increased the number of predictor variables until the best predictive model was obtained. Collinearity diagnosis was also performed by checking tolerance and variance inflation factor (VIF). Statistical analyses were conducted using SPSS Statistics 22 (SPSS Inc., Chicago, IL, United States).

## Results

### Subjects Characteristics

Three WD patients with T1 hyperintensities in the lentiform nucleus were excluded. A total of 17 neurological WD patients (10 males and 7 females) and 20 age-matched HC (12 males and 8 females) were included for further analysis. The mean age of WD patients and HC was 32.23 ± 7.55 (range 20–48) years and 30.4 ± 7.59 (range 18–43) years, respectively. There were no statistically differences in age distribution between the two groups (*P* = 0.478). The mean disease duration (from symptom onset to the time of MRI scans) was 10.83 ± 6.74 (range 0.5–21) years, and the mean treatment duration was 8.15 ± 6.07 (range 0.5–20) years. Four WD patients were treated with D-penicillamine and 13 WD patients were treated with DMSA.

T2/FLAIR hyperintensities in lentiform nucleus, Th, midbrain, and tegmentum of pons were the most common findings and were observed in 15 of 17 (88%) WD patients (Supplementary Figure S2). Brain atrophy was observed in 11 of 17 (65%) WD patients. Demographic and clinical characteristics of all participants are summarized in Table 1.

**TABLE 1:** Demographic and clinical characteristics of all subjects.

	WD patients (*N* = 17)	HC (*N* = 20)
Gender (male/female)	10/7	12/8
Age (years)*	32.23(7.55)	30.4 (7.59)
Disease duration (years)	10.83(6.74)	–
Treatment duration (years)	8.15 (6.07)	–
Kayser-Fleischer rings	16/17	–
Serum ceruloplasmin (<0.15 g/L)	17/17	–
UWDRS neurological subscores	13.71(12.14)	–
T2/FLAIR hyperintensities	15/17	0/20
Brian atrophy	11/17	0/20

Standard deviations are in parentheses. *P = 0.478; Mann-Whitney U test was used for age comparison. Normal reference range: serum ceruloplasmin, 0.2–0.45 g/L. FLAIR, fluid-attenuated inversion recovery; HC, healthy controls; N, number; UWDRS, Unified Wilson’s Disease Rating Scale; WD, Wilson’s disease.

### Susceptibility and Volume Analysis

**FIGURE 2:**
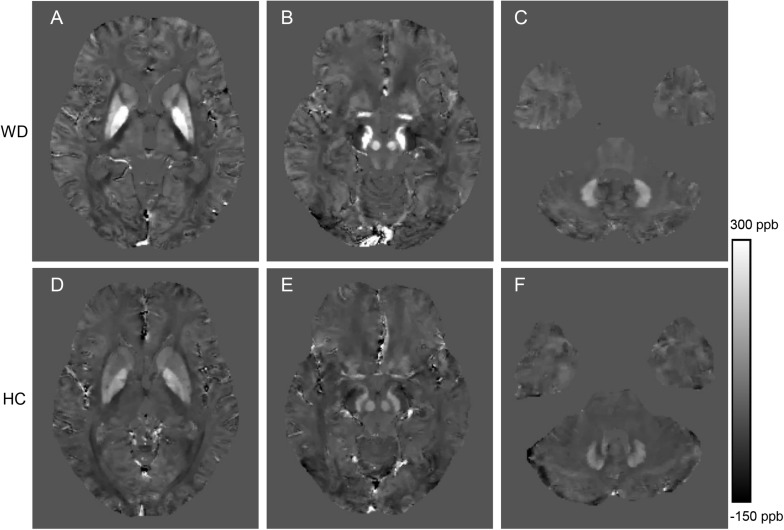
Comparison of QSM images from a WD patient (36 years old, male, **A–C**) and a healthy control (38 years old, male, **D–F**). The QSM images **(A,D)** include head of the caudate nucleus, globus pallidus, putamen and thalamus. The QSM images **(B,E)** include the red nucleus and substantia nigra. The QSM images **(C,F)** include the dentate nucleus. Significantly increased susceptibility was observed in the deep gray matter nuclei of the WD patient.

**FIGURE 3:**
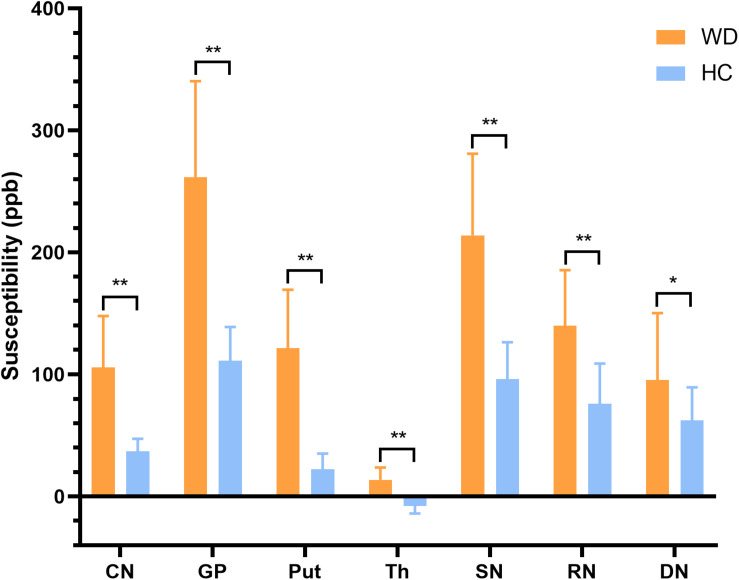
Comparison of mean susceptibility values of DGM between WD patients and HC. Susceptibility values of all examined DGM were higher in WD patients than in HC. *P < 0.05, and **P < 0.01. Error bars indicate standard deviation. CN, head of the caudate nucleus; DN, dentate nucleus; GP, globus pallidus; Put, putamen; RN, red nucleus; SN, substantia nigra; Th, thalamus.

**FIGURE 4:**
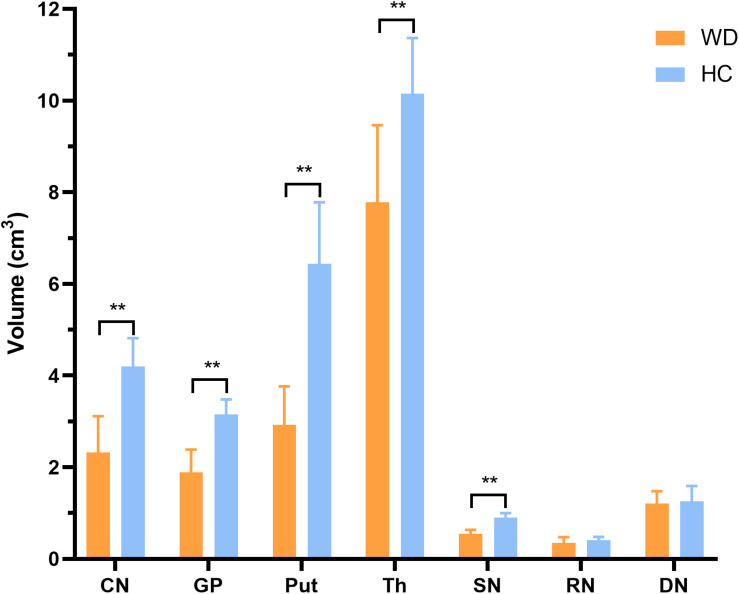
Comparison of mean volumes of DGM between WD patients and HC. Volume reductions were observed in CN, GP, Put, Th, and SN in WD patients. **P < 0.01. Error bars indicate standard deviation. CN, head of the caudate nucleus; DN, dentate nucleus; GP, globus pallidus; Put, putamen; RN, red nucleus; SN, substantia nigra; Th, thalamus.

In visual observation, high signals (increased susceptibility) could be observed in the DGM of WD patients. GP, Put, and SN had the most significant susceptibility changes in WD patients compared to HC. In addition, DGM atrophy was a common imaging feature of WD patients (Figure 2). The mean susceptibility values of all examined DGM in WD patients were higher than those in HC. Significantly increased susceptibility values were found in CN, GP, Put, Th, SN, and RN in WD patients (*P* < 0.001). The susceptibility value of DN was slightly higher in WD patients than in HC (*P* = 0.031) (Figure 3). Volume reductions were found in CN, GP, Put, Th, and SN in WD patients (*P* < 0.001), but the volumes of RN (*P* = 0.114) and DN (*P* = 0.614) were not significantly changed (Figure 4). More information about the susceptibility and volumes of DGM can be found in Supplementary Table S1.

### Correlation Between Susceptibility Values and Volumes of DGM

**FIGURE 5:**
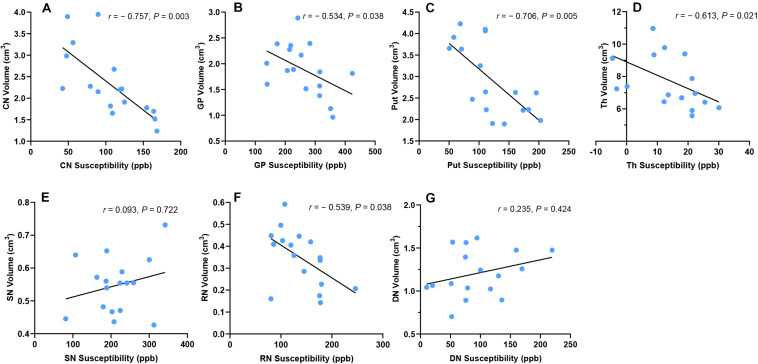
Correlation between susceptibility values and volumes of DGM in WD patients. Susceptibility values and volumes of DGM were negatively correlated in CN **(A)**, Put **(B)**, GP **(C)**, Th **(D)**, and RN **(F)**. No such correlation was found in SN **(E)** and DN **(G)**. CN, head of the caudate nucleus; DN, dentate nucleus; GP, globus pallidus; Put, putamen; RN, red nucleus; SN, substantia nigra; Th, thalamus.

In WD patients, susceptibility values and volumes of DGM were negatively correlated in CN (*r* = −0.757, *P* = 0.003), GP (*r* = −0.534, *P* = 0.038), Put (*r* = −0.706, *P* = 0.005), Th (*r* = −0.613, *P* = 0.021), and RN (*r* = −0.539, *P* = 0.038). No such correlation was found in SN (*r* = 0.093, *P* = 0.722) and DN (*r* = 0.235, *P* = 0.424) (Figure 5). After controlling for age, gender, and disease duration, susceptibility values and volumes of DGM were still negatively correlated in CN (*r*_*p*_ = −0.657, *P* = 0.037), Put (*r*_*p*_ = −0.667, *P* = 0.037), and Th (*r*_*p*_ = −0.613, *P* = 0.046). No correlation was found between susceptibility values and volumes of DGM in GP (*r*_*p*_ = −0.502, *P* = 0.118), SN (*r*_*p*_ = 0.063, *P* = 0.829), RN (*r*_*p*_ = −0.457, *P* = 0.141), and DN (*r*_*p*_ = 0.239, *P* = 0.478).

In the group of HC, there was no correlation between susceptibility values and volumes of DGM in CN (*r* = −0.302, *P* = 0.308), GP (*r* = −0.287, *P* = 0.308), Put (*r* = −0.577, *P* = 0.056), Th (*r* = −0.005, *P* = 0.985), SN (*r* = −0.477, *P* = 0.119), RN (*r* = −0.006, *P* = 0.985), and DN (*r* = 0.414, *P* = 0.163). Similar results were obtained after controlling for age and gender: CN (*r*_*p*_ = −0.313, *P* = 0.288), GP (*r*_*p*_ = −0.275, *P* = 0.315), Put (*r*_*p*_ = −0.313, *P* = 0.288), Th (*r*_*p*_ = −0.018, *P* = 0.945), SN (*r*_*p*_ = −0.381, *P* = 0.278), RN (*r*_*p*_ = −0.464, *P* = 0.256), and DN (*r*_*p*_ = 0.433, *P* = 0.256).

### Correlation Between Susceptibility Values of DGM and Neurological Impairment

**FIGURE 6:**
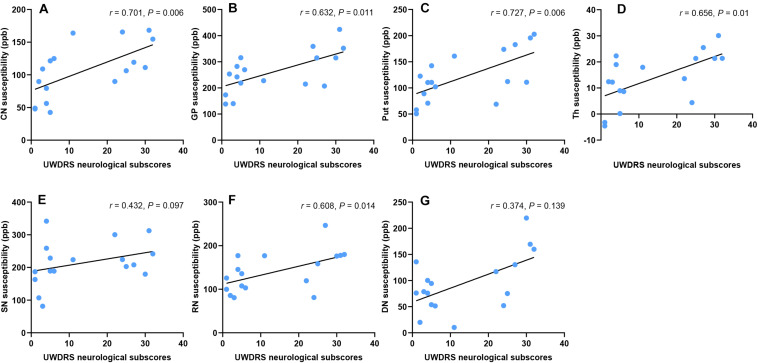
Correlation between susceptibility values of DGM and UWDRS neurological subscores. Susceptibility values were positively correlated with UWDRS neurological subscores in CN **(A)**, GP **(B)**, Put **(C)**, Th **(D)**, and RN **(F)**. No such correlation was found in SN **(E)** and DN **(G)**. CN, head of the caudate nucleus; DN, dentate nucleus; GP, globus pallidus; Put, putamen; RN, red nucleus; SN, substantia nigra; Th, thalamus.

Unified Wilson’s Disease Rating Scale neurological subscale was used to assess the severity of neurological impairment in WD patients, with a higher score indicating a more severe condition. The susceptibility values of DGM were positively correlated with UWDRS neurological subscores in CN (*r* = 0.701, *P* = 0.006), GP (*r* = 0.632, *P* = 0.011), Put (*r* = 0.727, *P* = 0.006), Th (*r* = 0.656, *P* = 0.01), and RN (*r* = 0.608, *P* = 0.014) in WD patients. No such correlation was found in SN (*r* = 0.432, *P* = 0.097) and DN (*r* = 0.374, *P* = 0.139) (Figure 6). After controlling for age, gender, and disease duration, susceptibility values of DGM were positively correlated with UWDRS neurological subscores in all examined DGM: CN (*r*_*p*_ = 0.669, *P* = 0.016), GP (*r*_*p*_ = 0.616, *P* = 0.026), Put (*r*_*p*_ = 0.76, *P* = 0.006), Th (*r*_*p*_ = 0.72, *P* = 0.009), SN (*r*_*p*_ = 0.588, *P* = 0.03), RN (*r*_*p*_ = 0.766, *P* = 0.006), and DN (*r*_*p*_ = 0.58, *P* = 0.03).

The multiple regression model revealed that the susceptibility values of Put, CN, and DN could well predict UWDRS neurological subscores. The susceptibility value of Put was superior to that of other DGM nuclei in predicting the UWDRS neurological subscores. In the model with only the susceptibility value of Put as a predictor variable, UWDRS neurological subscores increased by 1 point for every 6 ppb increase in Put (*P* = 0.005; *R*^2^ = 0.415). The model using the susceptibility values of CN and DN as predictor variables had the best ability in predicting the UWDRS neurological subscores. UWDRS neurological subscores increased by 1 point for every 6 ppb increase in CN (*P* < 0.001) or every 8 ppb increase in DN (*P* = 0.001; *R*^2^ = 0.745). Details about the multiple regression model can be found in Supplementary Table S2.

### Correlation Between Volumes of DGM and Neurological Impairment

There was no correlation between volumes of DGM and UWDRS neurological subscores in CN (*r* = −0.432, *P* = 0.146), GP (*r* = −0.625, *P* = 0.051), Put (*r* = −0.311, *P* = 0.262), Th (*r* = −0.513, *P* = 0.082), SN (*r* = −0.549, *P* = 0.079), RN (*r* = −0.367, *P* = 0.206), and DN (*r* = 0.205, *P* = 0.43). After controlling for age, gender, and disease duration, a negative correlation was found between the volume of Th and UWDRS neurological subscores (*r*_*p*_ = −0.689, *P* = 0.045). No such correlation was found in CN (*r*_*p*_ = −0.331, *P* = 0.289), GP (*r*_*p*_ = −0.575, *P* = 0.11), Put (*r*_*p*_ = −0.335, *P* = 0.289), SN (*r*_*p*_ = −0.502, *P* = 0.118), RN (*r*_*p*_ = −0.503, *P* = 0.118), and DN (*r*_*p*_ = 0.234, *P* = 0.42).

## Discussion

In this cross-sectional study, we found that the group average susceptibility values of all examined DGM in neurological WD patients were significantly higher than those in HC. Volume reductions were observed in CN, GP, Put, Th, and SN of WD patients, but RN in the midbrain and DN in the cerebellum were not affected. Susceptibility values and volumes of DGM were negatively correlated in CN, Put, and Th. In addition, increased DGM susceptibility was associated with high UWDRS neurological subscores in all examined DGM. There was only a weak correlation between volumes of DGM and UWDRS neurological subscores in Th. The results of our study were consistent with the theory that iron and copper accumulation in DGM was a character of neuropathology in neurological WD patients. To our knowledge, our study provided the first *in vivo* evidence that metal accumulation in the DGM was associated with DGM atrophy and neurological impairment in neurological WD patients.

Studies using traditional GRE MRI sequences such as T2^∗^-weighted images and SWI had identified iron accumulation in the brains of WD patients (Yang et al., 2015; Dusek et al., 2017). However, traditional GRE MRI sequences always suffer from blooming artifacts and the non-local effect (Li et al., 2012). Most importantly, traditional GRE MRI sequences cannot quantify susceptibility source without deconvolution, and phase changes cannot reflect the actual tissue susceptibility (Wang et al., 2017). QSM is a recently developed GRE MRI post-processing technique, which can quantitatively measure tissue susceptibility and is less affected by the external magnetic field (de Rochefort et al., 2010; Wang et al., 2017). Given iron content is about 3-fold higher than copper content in the DGM of neurological WD patients and the strong paramagnetism of iron species (Dusek et al., 2017; Dezortova et al., 2019), we attribute the increased susceptibility of DGM primarily to iron accumulation. However, we cannot exclude the influence of copper accumulation on susceptibility changes. In our study, significantly increased susceptibility was detected in all examined DGM nuclei, indicating metal accumulation in the DGM is common neuropathological feature of neurological WD patients. The highest susceptibilities values were observed in GP and SN, which was consistent with the distribution of iron species in neurological WD patients (Dusek et al., 2017). Whether iron species accumulate in the dentate nucleus in WD patients is controversial. Several postmortem studies analyzed the iron content in DN in WD patients, but the results were quite different (Litwin et al., 2013b; Dusek et al., 2017). We found that the mean susceptibility value of DN in WD patients was slightly higher than that in HC. The results of our study suggested that iron species tended to accumulate in the basal ganglia and brain stem of WD patients, while DN in the cerebellum was less affected.

Interestingly, increased susceptibility was also observed in Th, which contains large amount of white matter fiber tracts. Myelin in white matter is diamagnetic, and the increased susceptibility in Th may be caused by demyelination besides metal accumulation (Hametner et al., 2018; Dezortova et al., 2019). Demyelination is a common pathological feature of white matter in WD patients. Recent studies also revealed microstructure changes in Th and white matter of WD patients using diffusion tensor imaging (DTI) (Li et al., 2014; Dong et al., 2019). However, we cannot determine whether metal accumulation or demyelination is the main factor leading to increased susceptibility in Th. In addition, an increase in the mean bulk susceptibility of DGM may be induced by DGM atrophy. Metal concentration increases with tissue atrophy, but it is unclear whether the total metal content increases (Hernandez-Torres et al., 2019). Increased susceptibility was also observed in the DGM without atrophy (RN and DN) in WD patients. Therefore, we speculate that paramagnetic metal accumulation occurs in the DGM of WD patients. Histopathological studies are needed to confirm whether there is paramagnetic metal accumulation in the DGM of WD patients.

Brain atrophy is a common MRI feature of neurological WD patients and is associated with the severity of the neurological impairment (Smolinski et al., 2019; Dusek et al., 2020). Brain atrophy seems to be an irreversible process, but the neurological symptoms of WD patients are partly reversible after anti-copper treatment. Therefore, brain volume may be more appropriate as an assessment for chronic treated WD patients than as a marker for treatment monitoring. In WD patients, basal ganglia and brainstem are the most vulnerable regions, especially Put, which is a softening, brownish, atrophic nuclei with cavitations at macroscopic examination (Poujois et al., 2017). In this study, we found significant DGM atrophy in CN, GP, Put, Th, and SN, but RN and DN were not affected. Furthermore, we found that susceptibility values and volumes of DGM were negatively correlated in CN, Put, and Th, suggesting that metal accumulation in the DGM was associated with neuronal loss. The results of our study were consistent with the study of Dusek et al. (2017) which found Put atrophy was accompanied with increased iron content and iron-containing macrophages. However, it is not clear whether iron accumulation was merely a biomarker of neurodegeneration or a cause or contributor to the neurodegeneration itself (Yan et al., 2018). The advantage of QSM in measuring DGM volumes is that it can show the boundaries of DGM more clearly than traditional MRI sequences, which can ensure the accuracy of segmentation (Liu et al., 2013). To our knowledge, this study is the first one to investigate the DGM volume changes in WD using QSM.

Brain atrophy and iron accumulation is a common feature of neurodegenerative diseases such as Alzheimer’s disease and Parkinson’s disease (Lane et al., 2018; Jiang et al., 2019). Increased iron accumulation in the DGM may be explained by the influx of iron-containing phagocytic cells (macrophages), mitochondria damage and energy production failure, or decreased iron efflux due to ceruloplasmin dysfunction (Dusek et al., 2017, 2018). Ceruloplasmin is a ferroxidase that plays a critical role in maintaining iron homeostasis and preventing the formation of free radicals (Wang and Wang, 2019). Decreased serum ceruloplasmin may aggravate iron accumulation in WD, which is similar to the pathogenesis of aceruloplasminemia (Piperno and Alessio, 2018). As mentioned above, iron accumulation may be caused by neurodegeneration in WD, and iron accumulation also exacerbates neurodegeneration due to its redox properties.

We assessed the severity of neurological impairment of WD patients using UWDRS neurological subscale, and found that the susceptibility values of DGM were positively correlated with the severity of neurological impairment in all examined DGM. The correlation between iron accumulation and neuropathological changes of Put has been confirmed by a histopathological study (Dusek et al., 2017). Brain atrophy and brain MRI abnormalities reflecting iron accumulation, such as T2^∗^ and SWI hypointensities, were proposed as a part of imaging scale to assess the severity of MRI changes in WD. These chronic brain abnormalities were considered irreversible overtime, and showed positive correlation with the severity of neurological impairment (Dusek et al., 2020). Our study provided *in vivo* evidence that metal accumulation in the DGM was associated with DGM atrophy and neurological impairment in neurological WD patients. In addition, we found the susceptibility values of Put, CN, and DN could well predict the severity of neurological impairment. The susceptibility value of Put was superior to that of other DGM nuclei in predicting the UWDRS neurological subscores. The model using the susceptibility values of CN and DN as predictor variables had the best ability in predicting the UWDRS neurological subscores.

Few studies focused on the correlations between DGM atrophy and severity of neurological impairment in WD. Zou et al. (2019) reported that the volumes of bilateral Put and left GP were associated with the severity of neurological impairment in WD, which was assessed using modified Young scale. However, in this study, we only found a weak correlation between the volumes of DGM and the UWDRS neurological subscores in Th. The method of DGM volume assessment (manual or automatic segmentation), the selection of neurological scales, the small number of WD patients, and the high heterogeneity of WD patients may be the reasons for different results of the two studies. More studies are needed to determine the relationship between DGM atrophy and the severity of neurological impairment in WD. Compared with the volumes of DGM, the susceptibility values of DGM showed better correlations with UWDRS neurological subscores. The results of our study suggested that the susceptibility values of DGM could be used as a biomarker to assess the severity of neurodegeneration, which would be helpful in the diagnosis of WD, treatment monitoring, and as a surrogate outcome for clinical trials.

Several limitations in this study have to be mentioned. In some WD patients, DGM atrophy and local high susceptibility make the boundaries of DGM unclear, which may affect the accuracy of DGM segmentation. WD patients included in this study were treated patients with mild or moderate neurological symptoms. Patients with severe neurological impairment were excluded because they could not cooperate or remain still during MRI scans. It is unclear whether the susceptibility of DGM is still associated with neurological impairment in patients with severe neurological symptoms or in untreated patients. Due to limited sample size, the results of our study need to be confirmed in a larger cohort. In addition, a longitudinal study is required to determine whether the susceptibility of DGM changes with disease progression or symptom improvement.

In conclusion, our study found paramagnetic metal accumulation in the DGM of neurological WD patients, and provided *in vivo* evidence that metal accumulation in the DGM was associated with DGM atrophy and neurological impairment. The susceptibility of DGM could be used as a biomarker to assess the severity of neurodegeneration in WD.

## Data Availability Statement

The raw data supporting the conclusions of this article will be made available by the authors, without undue reservation.

## Ethics Statement

The studies involving human participants were reviewed and approved by Medical Ethics Committee of Tongren Hospital, Shanghai Jiao Tong University School of Medicine. The patients/participants provided their written informed consent to participate in this study.

## Author Contributions

X-ZY and J-LC developed the conception and design of the study, collected clinical and imaging data, and analyzed and interpreted the data. G-YL analyzed and interpreted the data, and revised the manuscript. J-QL and X-PW developed the conception and design of the study, and revised the manuscript. X-ZY wrote the first draft, and all of the authors critically reviewed the manuscript. All authors contributed to the article and approved the submitted version.

## Conflict of Interest

The authors declare that the research was conducted in the absence of any commercial or financial relationships that could be construed as a potential conflict of interest.

## Footnotes

1 http://www.itk-snap.org
