# Supplementary material for: Paramagnetic Metal Accumulation in the Deep Gray Matter Nuclei Is Associated With Neurodegeneration in Wilson’s Disease
Source: Front Neurosci. 2020 Sep 16;14:573633. doi: 10.3389/fnins.2020.573633 (PMC7525019; doi:10.3389/fnins.2020.573633)
Supplement: Supplementary file 1 [file Data_Sheet_1.pdf]

Supplementary Materials

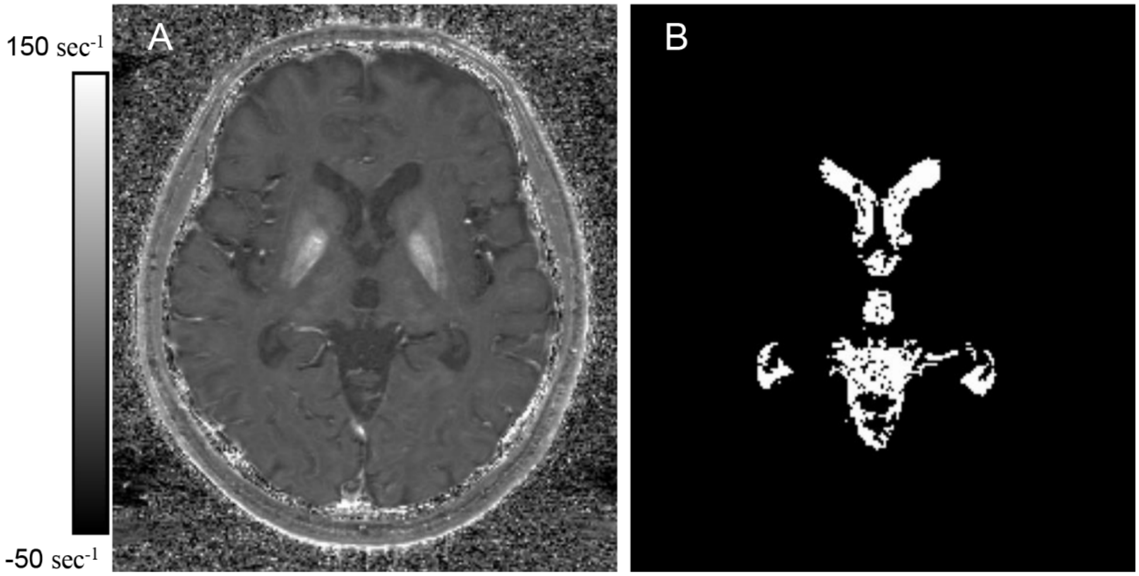

Figure 1 Example images showing R2* map and CSF mask (WD patient, 36 years old, male). R2* map (**A**) was used to automatically determine the ventricular CSF mask M_{CSF} (**B**) using MEDI+0 algorithm.

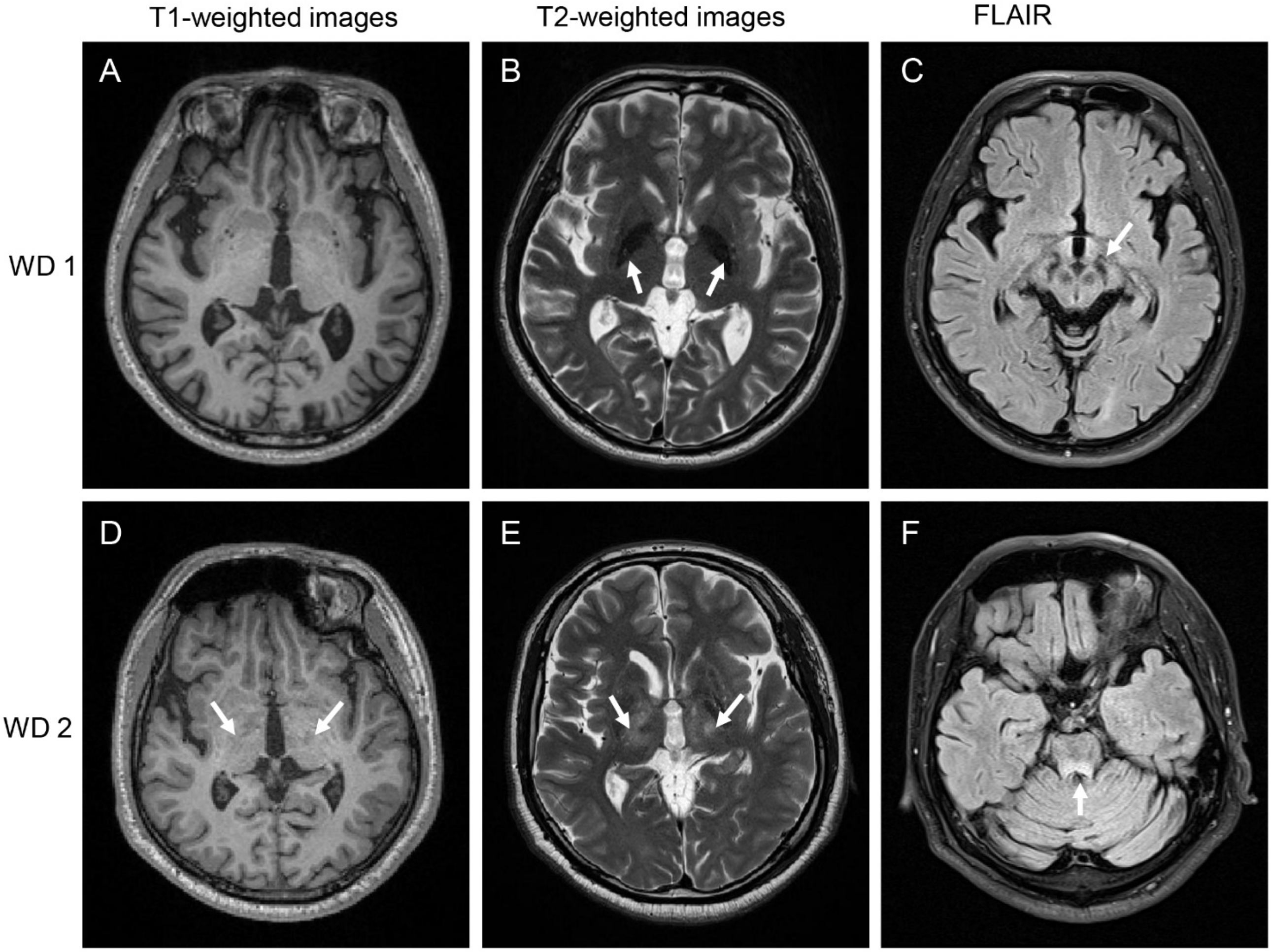

Figure 2 Conventional MRI abnormalities in neurological WD patients (WD 1: 36 years old, male; WD 2: 33 years old, male). The abnormalities were marked with white arrows. (**A**) normal T1-weighted images. (**B**) T2 hypointensities in the globus pallidus. (**C**) the sign of "face of giant panda" in the midbrain in FLAIR images. (**D**) T1 hypointensities in the lenticular nucleus and thalamus. (**E**) T2-hyperintensities in the lenticular nucleus and thalamus. (**F**) hyperintensities in the tegmentum of pons in FLAIR images. FLAIR, fluid-attenuated inversion recovery; WD, Wilson's disease.

Table 1 The susceptibility values and volumes of DGM in all subjects

	Susceptibility values (ppb)			Volumes (cm^3)		
	WD (N = 17)	HC (N = 20)	*P*-value*	WD (N = 17)	HC (N = 20)	*P*-value*
CN	105.79 (42.08)	36.82 (10.4)	< 0.001	2.321 (0.793)	4.2 (0.618)	< 0.001
GP	261.52 (78.84)	111.26 (27.58)	< 0.001	1.885 (0.499)	3.156 (0.326)	< 0.001
Put	121.39 (48.01)	22.16 (12.89)	< 0.001	2.922 (0.845)	6.438 (1.343)	< 0.001
Th	13.6 (10.14)	− 7.83 (6.09)	< 0.001	7.78 (1.684)	10.151 (1.217)	< 0.001
SN	213.96 (66.92)	96.00 (30.42)	< 0.001	0.547 (0.085)	0.898 (0.1)	< 0.001
RN	139.8 (45.65)	75.98 (33.01)	< 0.001	0.346 (0.129)	0.409 (0.07)	0.114
DN	95.23 (54.95)	62.39 (27.1)	0.031	1.206 (0.272)	1.254 (0.337)	0.614

Standard deviations are in parentheses.

* P-values were corrected by controlling the false discovery rate at a level of 0.05.

CN, head of the caudate nucleus; DGM, deep gray matter; DN, dentate nucleus; GP, globus pallidus; N, number; Put, putamen; RN, red nucleus; SN, substantia nigra; Th, thalamus; WD, Wilson's disease.

Table 2 Stepwise multiple regression models using susceptibility values of DGM to predict UWDRS neurological subscores

Model	R^2	Adjusted R^2	Model *P*-value	Predictor	Unstandardized Coefficient	Standardized Coefficient	*P*-value
Model 1	0.415	0.376	0.005	Put	0.163 (0.05)	0.644	0.005
Model 2	0.678	0.632	< 0.001	Put	0.146 (0.039)	0.577	0.002
				DN	0.114 (0.034)	0.517	0.004
Model 3	0.774	0.722	< 0.001	Put	0.063 (0.049)	0.248	0.22
				DN	0.123 (0.03)	0.555	0.001
				CN	0.13 (0.055)	0.45	0.035
Model 4	0.745	0.709	< 0.001	CN	0.181 (0.039)	0.628	< 0.001
				DN	0.129 (0.03)	0.585	0.001

Standard errors are in parentheses.

$P < 0.05$ was considered significant. CN, head of the caudate nucleus; DGM, deep gray matter; DN, dentate nucleus; Put, putamen
